# Understanding the socio-demographic and programmatic factors associated with adolescent motherhood and its association with child undernutrition in Bangladesh

**DOI:** 10.1186/s12889-024-19355-3

**Published:** 2024-08-13

**Authors:** Md. Alamgir Hossain, Novel Chandra Das, Md. Tariqujjaman, Abu Bakkar Siddique, Rubaiya Matin Chandrima, Md. Fakhar Uddin, S. M Hasibul Islam, Abu Sayeed, Anisuddin Ahmed, Shams El Arifeen, Hassan Rushekh Mahmood, Ahmed Ehsanur Rahman, Aniqa Tasnim Hossain

**Affiliations:** 1 Maternal and Child Health Division (MCHD), International Centre for Diarrheal Disease Research, Bangladesh (icddr,b), 68, Shaheed Tajuddin Ahmed Sarani, Mohakhali, Dhaka, 1212 Bangladesh; 2 Health System and Population Studies Division (HSPSD), International Centre for Diarrheal Disease Research, Bangladesh (icddr,b), 68, Shaheed Tajuddin Ahmed Sarani, Mohakhali, Dhaka, 1212 Bangladesh; 3 Nutrition Research Division (NRD), International Centre for Diarrheal Disease Research, Bangladesh (icddr,b), 68, Shaheed Tajuddin Ahmed Sarani, Mohakhali, Dhaka, 1212 Bangladesh; 4 Global Health and Migration Unit, Department of Women’s and Children’s Health, Uppsala University, Uppsala, Sweden; 5 NIHR Global Health Research Unit on Respiratory Health (RESPIRE), Usher Institute, University of Edinburgh, Edinburgh, UK

**Keywords:** Adolescent mothers, Adolescent motherhood, First childbirth, Child undernutrition, Programmatic gaps, Bangladesh

## Abstract

**Background:**

Worldwide, a significant number of girls become mothers during adolescence. In Bangladesh, adolescent childbirth is highly prevalent and has adverse effects on children’s health and undernutrition. We aimed to identify the relationship between the undernutrition of children and adolescent motherhood, the factors associated with adolescent mothers’ age at first birth, and to examine the programmatic factors and gaps influencing children’s undernutrition in Bangladesh.

**Methods:**

We analysed the ‘Bangladesh Demographic and Health Survey’ BDHS-17-18 data and desk review. To examine the factors associated with adolescent motherhood and its impact on child undernutrition, data from 7,643 mother-child pairs were selected. Child stunting, wasting, and underweight were measured according to the World Health Organisation (WHO) median growth guidelines based on z-scores − 2. Univariate, bivariate, simple, and multiple logistic regressions were used for analyse. We followed the systematic procedures for the literature review.

**Results:**

Approximately, 89% of adolescents aged ≤ 19 years were married and 71% of them gave their first childbirth. Children of adolescent mothers (≤ 19 years) were significantly 1.68 times more wasted (aOR: 1.68; 95% CI: 1.08 to 2.64), 1.37 times more underweight (aOR: 1.37; 95% CI: 1.01 to 1.86) and either form 1.32 times more stunting, wasting or underweight (aOR:1.32; 95% Cl: 1.05 to 1.66) compared to the children of adult mothers (> 19 years) after adjusting potential confounders. The factors associated with mothers’ first childbirth during adolescence were the age gap between husband and wife 5–10 years (aOR: 1.81; 95% Cl: 1.57–2.10) and age gap > 10 years (aOR: 2.41; 95% Cl: 1.96–2.97) compared with the age group < 5 years, and husbands’ education (aOR: 1.29; 95% Cl: 1.04–1.61) compared with the uneducated husbands. In the literature review, we found potential gaps in focusing on the Adolescent Sexual and Reproductive Health (ASRH) program in Bangladesh, from thirty-two programmes only half of them focused on adolescents aged 10–19 years, and eleven programmes focused only on girls.

**Conclusion:**

Children of adolescent mothers are at risk of wasting, underweight, and any form of undernutrition. For effective policies and interventions in Bangladesh, it is important to emphasise delaying adolescent pregnancy and prioritising child undernutrition.

**Supplementary Information:**

The online version contains supplementary material available at 10.1186/s12889-024-19355-3.

## Background

Globally, in 2022 around 13% of adolescent girls gave birth, and in South Asia, the rate was around 10% [1]. Adolescent pregnancy is a global phenomenon with severe health, social, and economic consequences, is higher among people with low education and economic status [2]. In low- and middle-income countries (LMICs), it is predicted that every year around 21 million mothers aged 15–19 years become pregnant, with 12 million giving birth [2]. In Bangladesh, marriage before the eighteen years is not legal [3]. Despite this, it persists because of the level of education among partners, partners’ occupation, religious influences, geographical location, pressure from the family, dowries, social norms, poverty, family honour, threats of sexual violence, fear of technological harassment, and concerns about security and protection [4–8]. In 2017, Bangladeshi parliament authorised early marriages under some circumstances [9]. Consequently, many teenage girls married before they could legally marry [10].

There is a complex relationship between mother’s first childbirth during adolescence and child undernutrition. Around one in two teenage girls in South Asia married before becoming eighteen, and one in five of these girls gave birth to a child before reaching eighteen [11]. Children born to teenage mothers are at risk of being undernourished [12]. The birth outcomes associated with adolescent mothers include preeclampsia, anemia, sexually transmitted disease, low birth weight, preterm birth, stillbirth, neonatal and infant mortality, severe newborn diseases, and delayed growth [13–15]. A prospective study discovered that preterm birth, low birth weight, stunting, and inability to finish secondary school were all related to the age of adolescent mothers (≤ 19 years) [16]. The lack of appropriate feeding practices for newborns and teenagers is one of the many variables contributing to mother and child undernutrition in developing nations [17]. About 71% of new mothers initiate breastfeeding; however, the process is impeded by difficulties related to labor and delivery as well as a lack of social support [18]. Among teenage mothers, 36% breastfed their children within an hour [19]. Adolescent pregnancies are associated with negative health consequences for mothers and babies and are linked poor child survival and maternal and child mortality [20–22]. Around half of adolescent pregnancies were unintended, and neonatal deaths were twice as high as those of adult mothers [23]. Children born to the youngest teen mothers are significantly disadvantaged in being less likely to receive well-baby care, have less stimulating home environments, and have lower cognitive achievement scores compared to peers born to older mothers [24].

Child undernutrition has serious long-term implications for health and well-being. Worldwide in 2020, among under-five children, 150 million were stunted, 45 million were wasted [25] and 47 million were underweight [26]. In LMICs, undernutrition in childhood, such as stunting, wasting, and underweight, remains a crucial health problem. Approximately, 45% of under-five mortality in LMICs is caused by undernutrition [25]. From stunting and wasting around 1.8 million children pass away, and 12% of years are adjusted for disability every year in the world [27]. Undernourished children may exhibit neurodevelopmental impairments, abnormal behavior, poor performance in school, and mental health issues [28].

Multifaceted issues affect the prevalence of adolescent pregnancies in developing countries. The causes of teenage pregnancies in developing nations are poor, low parental education, and cultural influence [29]. Bangladesh has taken initiatives to reduce adolescent marriage, but it is still common in rural and remote areas. Marriage during adolescence impacts children’s health and nutrition, but limited evidence is available to examine it in detail. In this study, we identified the factors related to the child’s undernutrition and the mother’s age at first childbirth, associated factors of first childbirth during adolescence, and programmatic factors and gaps in the undernutrition of children of adolescent mothers in Bangladesh.

## Methodology

This analysis was performed based on nationally representative BDHS-17-18 data and a literature review following systematic procedures. To identify the factors associated with the child’s undernutrition and the mother’s age at first childbirth, and associated factors of first childbirth during the adolescent period, we used the BDHS-17-18 dataset. We used a desk-based literature review to examine programmatic factors and gaps in the undernutrition of children of adolescent mothers in Bangladesh. The analysis and literature review data were analyzed separately, triangulated, and combined in the results section.

### Data analysis

#### Data source

We extracted data from the BDHS-2017-18. The DHS website provides datasets that were downloaded after obtaining authorisation from the DHS program. The BDHS produces crucial national data on nutrition and maternal and child health [30].

#### Study population

**Fig. 1:**
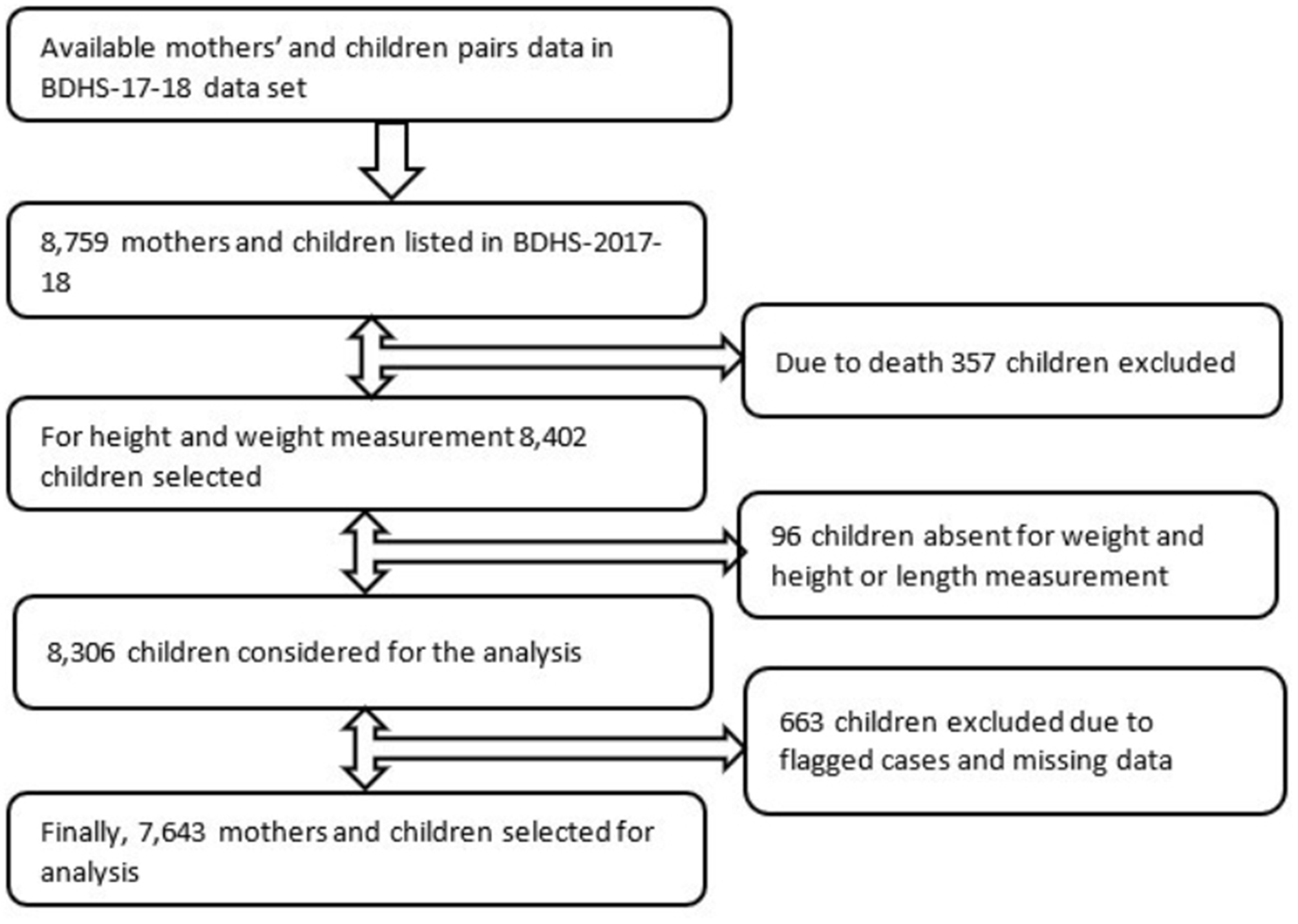
Sampling flow of mothers’ first childbirth during adolescence and child’s undernutrition

To determined the association between adolescent mothers’ first childbirth during adolescence and their child’s undernutrition, and to determine the factors associated with a mother’s first childbirth during adolescence, 7,643 mothers and child pairs were considered after eliminating missing data, height constraints, and labeled cases (inconsistent, inconsequential, and not applicable). In the BDHS-17-18 dataset, we found data from 8,759 mothers and children; of these 357 children were excluded due to death, 96 children were absent for weight, height, and length measurement, and 663 children were excluded due to flagged cases and missing data (Fig. 1). The BDHS 2017–18 report provides detailed information on the survey, including sampling strategy, sample size calculation, and data-gathering process [30].

#### Outcome variables

To explore the association of mothers aged ≤ 19 years during first childbirth with undernutrition in under-5 children, we followed the procedures for defining the undernutrition variables. The primary outcome variables were the child’s anthropometric measurements, such as stunting (height for age Z score), wasting (weight for height Z score), and underweight (weight for age Z score) for calculation of age - and sex-specific child growth guidelines based on z-scores below − 2 [31].

We categorized the variables between ≤ 19 years and > 19 years as adolescents age is 10–19 years to determine mothers’ first childbirth during adolescence.

#### Independent and exposure variables

To find the relationship between mother’s first childbirth during adolescent motherhood and the undernutrition of their children, we considered the independent factors including mothers’ age during first childbirth (≤ 19, > 19) years, age of marriage (adolescent age ≤ 19, older age > 19) years, mothers’ BMI (underweight < 18.5 to 18.5 kg/m2, normal weight ≥ 18.6 to 24.9 kg/m2, overweight and obesity 25 to > 29.9 kg/m2), fathers’ and mothers’ education (no formal education, primary, secondary, and higher), ANC visits (≤ 4 times, > 4 times), delivery via C-section (yes, no), types of household heads (self, father, husband, fathers-in-law, and others), currently working status (working, not working), children age (≤ 24, > 24) months, sex of children (male, female) wealth index (poorest, poorer, middle, richer, richest), administrative division (Barishal, Rajshahi, Dhaka, Khulna, Sylhet, Mymensingh, Rangpur, Chattogram), childbirth weight (low birth weight < 2500 gm and normal weight ≥ 2500 gm).

To find out the associated factors of mothers’ first childbirth during adolescent motherhood, we considered the variables such as the ‘age gap with the husband’ (< 5 years, 5–10 years, > 10 years), ‘adolescent mothers’ employment’ (not working, working), mother’s and husband’s education (no formal education, primary, secondary, higher), media exposure (not at all, < once a week, once a week), place of residence (urban, rural), wealth index (poorest, poorer, middle, richer, richest), administrative division (Barishal, Rajshahi, Dhaka, Khulna, Sylhet, Mymensingh, Rangpur, Chattogram).

### Statistical analysis

Data were analysed using Stata version 15 [32]. Weighted percentages were calculated using descriptive statistics. We used bivariate analysis including the chi-square test and simple and multiple logistic regressions. For the relationship between mother’s first childbirth during adolescence and the undernutrition of their children, we used simple and multiple logistic regression analysis, after adjusting potential confounders such as child age, child sex, wealth index, mothers’ BMI, types of household heads, birth-weight of children, and working status of respondent based on the previous studies [12, 33–36]. Additionally, we portrayed a composite association for child undernutrition, where we categorized undernutrition as at least one under-nutritional status among stunting, wasting, or underweight.

Moreover, we illustrated a line chart to understand the distribution of age groups for ‘mother’s age at first birth’ and ‘age of marriage’ simultaneously. We considered significant factors using the bivariate chi-square test for multiple logistic regression to observe the association with mothers’ first childbirth during adolescence. In this study, a P value ≤ 0.05 was statistically significant.

### Literature review

#### Search criteria

To understand the gaps in programmatic focuses related to adolescent mothers’ and children’s undernutrition in Bangladesh, we conducted a literature review. For this review, we used search terms such as (“programme” OR “program” OR “project” OR “implementation” AND “adolescent mother” OR “young mother” OR “early mother” AND “adolescent pregnancy” OR “early pregnancy” OR “young pregnancy” AND “girls’ marriage” OR “early marriage” OR “child marriage” AND “birth” OR “births” OR “first births” OR “first childbirths” AND “child” OR “children” OR “child’s” AND “undernutrition” OR “undernourished” OR “undernourishment” AND “BD” OR “Bangladesh”). The search was conducted in Google Scholar, PubMed, and manually from existing literature.

#### Article selection

We considered any study design for the literature review, including published articles, literature reviews, grey literature, reports, strategic plans, and action plans from Bangladesh. Articles were eliminated based on the title, abstract, and full-text screening. We considered the literature published from January 2015 to December 2023. Pre-2015 articles were considered older evidence to explain the situation. Articles and reports published in the English Language were retrieved. In this literature review, we found 3,018 articles and 5 reports from databases, websites, citations, and organizational websites. We excluded 2,450 articles for unmatched titles and duplications. We excluded 480 articles as per the review of the abstract and those not in the English language. We excluded 60 articles not focus on adolescent mothers aged ≤ 19 years and their children’s undernutrition. Finally, 18 articles that did not represent Bangladesh or data before 2015 were excluded. We consider 10 articles and 5 reports for the literature review (Supplementary Fig. 1).

#### Ethical consideration

The Institutional Review Board at ICF (ICF provided technical assistance through the DHS Program, a USAID-funded project providing support and technical assistance in implementation of population and health surveys in countries worldwide) approved us to perform the analysis of BDHS-17-18 data. All participants provided their consent while data were collected [30].

## Results

Table 1 describes the background characteristics of the study participants of the mothers’ first childbirth during adolescence and the undernutrition of children. About 71% of mothers gave their first childbirth at an age below or equal to 19 years, 56% of heads of households were husbands, and 60% of mothers were in good health according to BMI. Approximately, 50% of mothers had finished their secondary education, whereas 35% of fathers had finished their primary education. About 35% of mothers give birth to their babies by Cesarean section. Moreover, 16% of children had a low birth weight (< 2500 gm) at birth, and 40% of the mothers had jobs.

**Table 1:** Background characteristics of study participants of mothers’ first childbirth during adolescence and their child’s undernutrition

Characteristics	Un-weighted number and percentage *N* (%)	Weighted percentage (%) (*N* = 7,643)
**Mother’s age during first childbirth**		
≤ 19 years	5,356 (70.1)	71.06
> 19 years	2,287 (29.9)	28.5
**Age of marriage**		
≤ 19 years	6702 (87.7)	89.26
> 19 years	941 (12.3)	10.74
**Mother’s BMI**		
Underweight	1,128 (14.8)	13.9
Normal weight	4,543 (59.5)	60.5
Overweight/ Obese	1,972 (25.7)	25.6
**Mother’s education**		
No formal education	537 (7.0)	7.0
Primary	2,209 (28.9)	28.6
Secondary	3,619 (47.4)	49.0
Higher	1,278 (16.7)	15.4
**ANC visits (** ***N*** ** = 4,531)**		
≤ 4 times visit	2,886 (63.7)	64.5
**Delivery via C-section (** ***N*** ** = 4,724)**		
No C-section	3,154 (66.8)	66.7
**Types of household head**		
Self	528 (6.9)	7.6
Husband	4,397 (57.5)	56.3
Father	885 (11.6)	11.9
Fathers-in-law	1,432 (18.7)	19.3
Others	401 (5.3)	4.9
**Father’s education**		
No formal education	1,140 (14.9)	14.9
Primary	2,630 (34.4)	34.7
Secondary	2,473 (32.4)	33.3
Higher	1,400 (18.3)	17.1
**Wealth index**		
Poorest	1,709 (22.4)	21.8
Poorer	1,544 (20.2)	20.4
Middle	1,377 (18.0)	19.1
Richer	1,528 (20.0)	20.3
Richest	1,485 (19.4)	18.4
**Currently working status**		
Yes	3,089 (40.4)	40.2
**Childbirth weight (** ***N*** ** = 2136)**		
Low birth weight (< 2500 gm)	328 (15.4)	15.7
Normal weight (≥ 2500 gm)	1,808 (84.6)	84.5
**Age of children (months)**		
≤ 24 (months)	3,253 (42.6)	42.6
**Sex of children**		
Male child	3,985 (52.1)	52.2

**Fig. 2:**
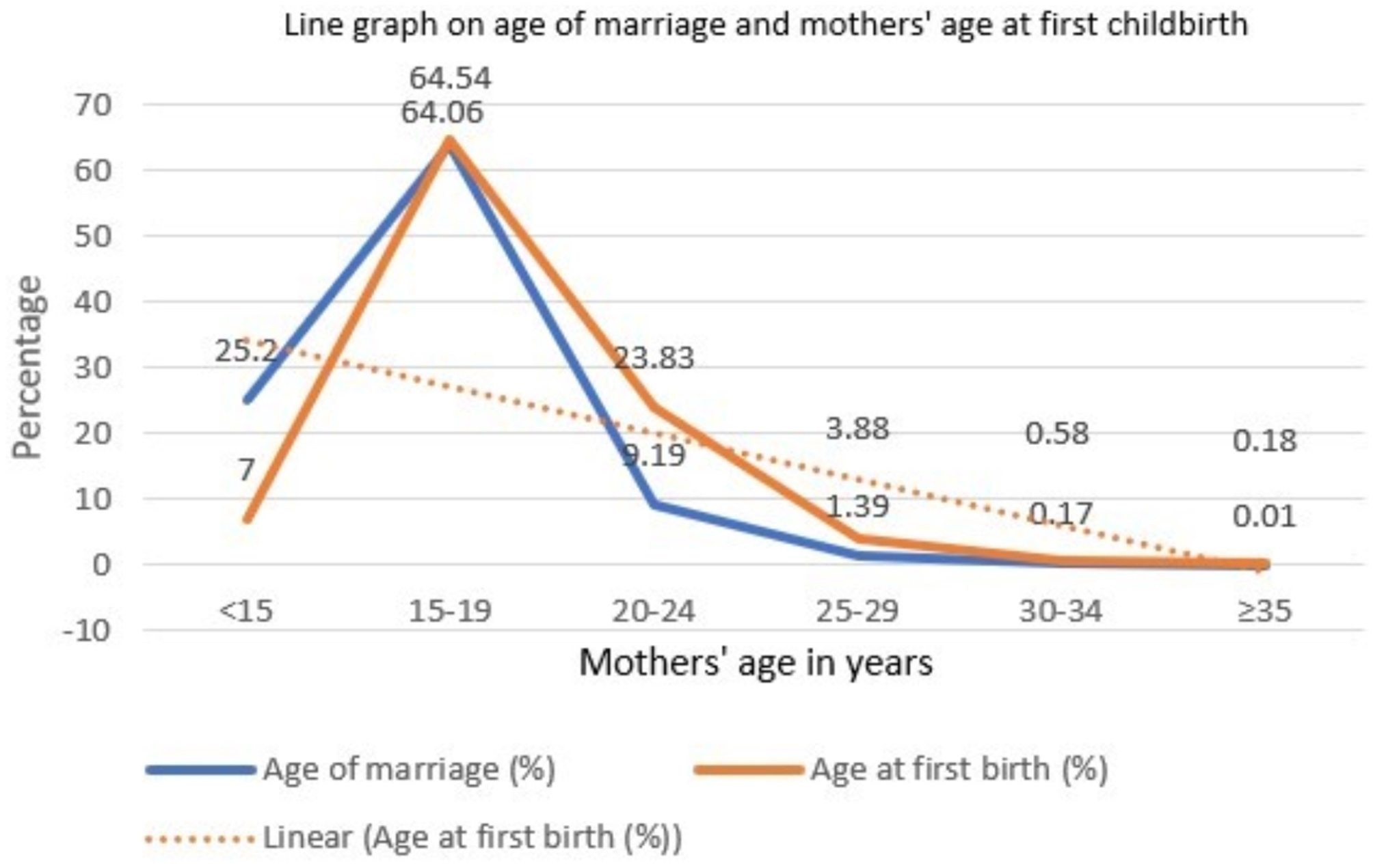
Line graph on age of marriage and mother’s age during first childbirth

Figure 2, demonstrates the line graph on the age of marriage and mothers’ age at first childbirth. 25% of mothers aged < 15 years conduct marriage and among them 7% give childbirth. 89% of mothers aged ≤ 19 years conduct marriage and among them, 71% of mothers have their first childbirth.

**Fig. 3:**
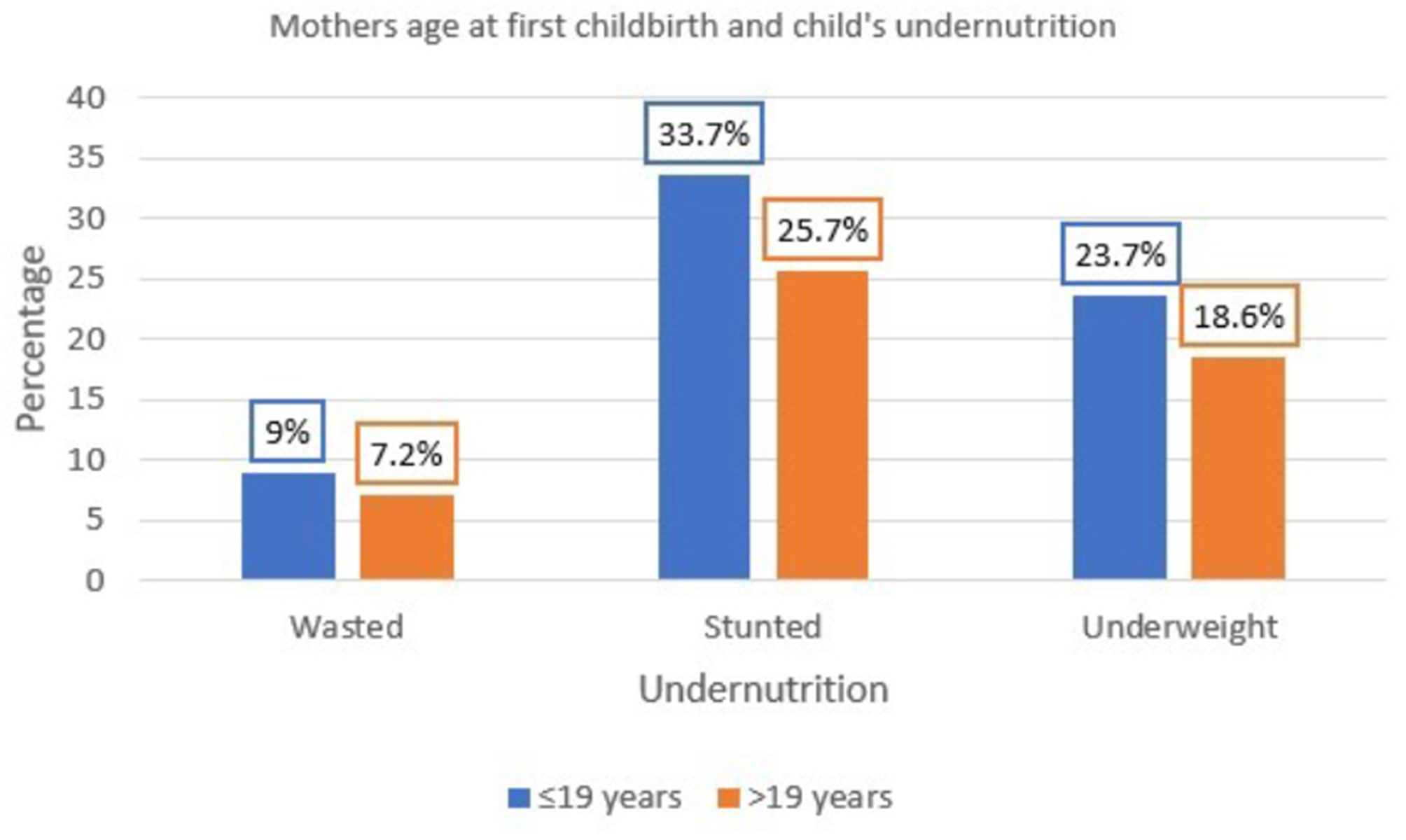
Association of adolescent mother age during first childbirth and child’s undernutrition

Figure 3, demonstrates the mother’s first childbirth during adolescence and the child’s undernutrition such as wasting at 9%, stunting at 34%, and underweight at 24% compared to the children of adult mothers’ who were wasted at 7%, stunted at 26%, and underweight at 19%.

Chi-square test results are mentioned in supplementary Table 1, which describes the association of the mother’s first childbirth (≤ 19 years, > 19 years) with their child’s undernutrition where we found the mother’s age during first childbirth, mother’s BMI, mother’s education, delivery via C-section, types of household head, currently working status, father’s education, wealth index, childbirth weight, age of children and sex of children were statistically significant (Supplementary Table 1).

The chi-square test result also mentioned in the supplementary Table 2, describes the associated factors of a mother’s first childbirth (adolescent mothers’ ≤19 and adult mothers > 19) years, where we found that the spousal age gap, mothers’ employment status, mothers and partners’ education level, media exposure, place of residence, wealth index, and administrative division were significant (Supplementary Table 2).

Table 2 demonstrates the relationship between the mother’s first childbirth during adolescence and their child’s undernutrition compared to adult mothers. Simple and multiple logistic regression models were applied to analysis, after adjusting for probable confounders. The results of the regression model showed that children born to mothers aged ≤ 19 years had 1.7 times higher chances of being wasted [Adjusted Odds Ratio: aOR] (aOR: 1.68, 95% CI: 1.08–2.64), and 1.4 times higher chances of being underweight (aOR: 1.37, 95% CI: 1.01–1.86) compared with mothers aged > 19 years. In composite analysis, we also found that children of adolescent mothers had either stunting or wasting or were underweight 1.3 times higher (aOR: 1.32, 95% Cl: 1.05–1.66) compared to the adult mothers’ (> 19 years). However, we found that stunted is 1.25 (aOR: 1.25: 95% Cl: 0.97–1.59) times higher risk where the P value is 0.08 for children of adolescent mothers compared with children of adult mothers (Table 2).

**Table 2:** Association between the mother’s first childbirth during adolescence with undernutrition of children

Characteristics	Unadjusted Odds Ratio (UOR) (95% CI)	*P* value	Adjusted^a^ Odds Ratio (aOR) (95% CI)	*P* value
**Wasting**				
> 19 years	Reference		Reference	
≤ 19 years	1.3 (1.03–1.61)	0.024*	1.68 (1.08–2.64)	0.02*
**Stunting**				
> 19 years	Reference		Reference	
≤ 19 years	1.4 (1.23–1.59)	< 0.001*	1.25 (0.97–1.59)	0.08
**Underweight**				
> 19 years	Reference		Reference	
≤ 19 years	1.3 (1.14–1.52)	< 0.001*	1.37 (1.01–1.86)	0.04*
Composite analysis**				
> 19 years	Reference		Reference	
≤ 19 years	1.4 (1.23–1.59)	< 0.001*	1.32 (1.05–1.66)	0.02*

^a^ Model was adjusted for child sex, child age, wealth index, mother’s BMI, birth weights of children, working status, and types of household head

∗Significant P values

**Either form of stunting, wasting, or underweight

Table 3 describes the determinants of mothers’ first childbirth during adolescence. In the regression model, we found that the factors associated with mothers’ first childbirth during adolescence were the age gap between the husband and wife five to ten years (aOR: 1.81; 95% Cl: 1.57–2.10) and the age gap more than ten years (aOR: 2.41; 95% Cl: 1.96–2.97) were significantly associated compared with the age group < 5 years, and primary education of the adolescent partners (aOR: 1.29; 95% Cl: 1.04–1.61) were significantly associated compared with the uneducated partners (Table 3).

**Table 3:** Associated factors of mother’s first childbirth during adolescence

Characteristics	Unadjusted Odds Tation (UOR) (95% CI)	*P* value	Adjusted Odds Ratio (aOR) (95% CI)	*P* value
**Age gap with husband**				
< 5 years	Reference		Reference	
5–10 years	1.59 (1.39, 1.83)	< 0.001	1.81 (1.57, 2.10)	< 0.001
> 10 years	1.71 (1.43, 2.05)	< 0.001	2.41 (1.96, 2.97)	< 0.001
**Adolescent mothers’ employment**				
Not working	Reference		Reference	
Working	1.19 (1.05, 1.35)	0.00	0.87 (0.76, 1.00)	0.055
**Husband’s education**				
No formal education	Reference		Reference	
Primary	1.09 (0.89, 1.34)	0.36	1.29 (1.04, 1.61)	0.018
Secondary	0.774 (0.63, 0.94)	0.00	1.17 (0.92, 1.48)	0.182
Higher	0.24 (0.19, 0.30)	< 0.001	0.64 (0.49, 0.85)	< 0.001
**Media exposure**				
Not at all	Reference		Reference	
< Once a week	0.81 (0.63, 1.04)	0.09	1.00 (0.77, 1.29)	0.96
Once a week	0.52 (0.45, 0.61)	< 0.001	0.86 (0.726, 1.02)	0.09
**Place of residence**				
Urban	Reference		Reference	
Rural	1.77 (1.50, 2.07)	< 0.001	1.18 (0.99, 1.40)	0.06
**Mother’s education**				
No formal education	Reference		Reference	
Primary	1.16 (0.88, 1.53)	0.28	1.16 (0.85, 1.57)	0.33
Secondary	0.90 (0.69, 1.17)	0.43	0.94 (0.69, 1.28)	0.71
Higher	0.17 (0.13, 0.23)	< 0.001	0.26 (0.18, 0.38)	< 0.001
**Wealth index**				
Poorest	Reference		Reference	
Poorer	0.80 (0.65, 0.98)	0.03	0.86 (0.69, 1.08)	0.21
Middle	0.56 (0.45, 0.69)	< 0.001	0.77 (0.55, 0.93)	0.01
Richer	0.44 (0.35, 0.55)	< 0.001	0.67 (0.50, 0.89)	0.00
Richest	0.23 (0.19, 0.29)	< 0.001	0.56 (0.40, 0.77)	0.00
**Administrative division**				
Barisal	Reference		Reference	
Chattogram	0.97 (0.74, 1.27)	0.84	1.01 (0.77,1.32)	0.91
Dhaka	0.66 (0.50, 0.87)	0.00	0.78 (0.58, 1.04)	0.10
Khulna	0.85 (0.64, 1.12)	0.23	0.98 (0.74, 1.31)	0.92
Mymensingh	0.90 (0.68, 1.19)	0.46	0.89 (0.68,1.17)	0.41
Rajshahi	1.09 (0.81, 1.47)	0.56	1.23 (0.923, 1.65)	0.15
Rangpur	1.18 (0.89, 1.59)	0.27	1.30 (0.978, 1.73)	0.070
Sylhet	0.62 (0.47, 0.82)	0.00	0.44 (0.33, 0.58)	< 0.001

### Understand the programme gaps in adolescent mothers’ and children’s undernutrition

**Fig. 4:**
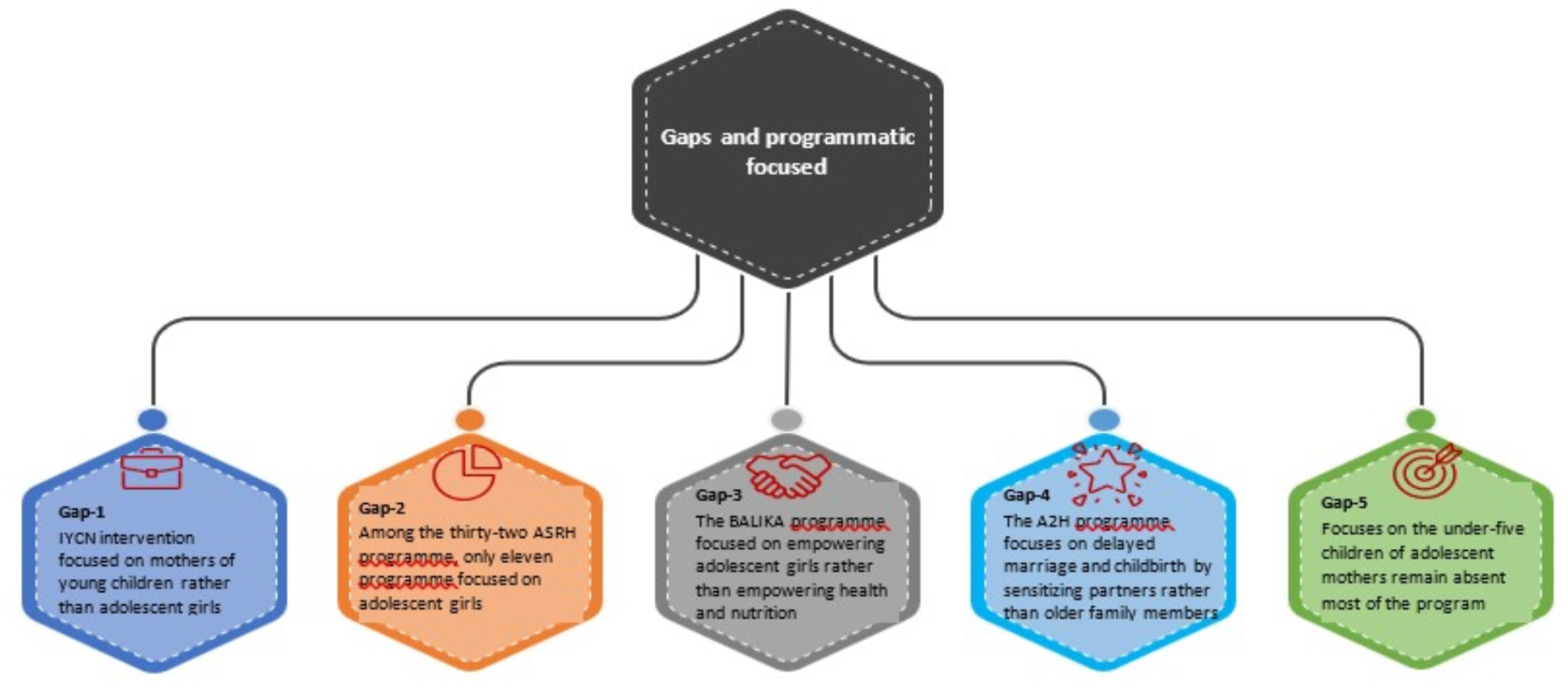
programmatic gaps and focus on adolescent mothers’ and children’s undernutrition

In Bangladesh, Infant and Young Child Nutrition (IYCN) interventions are typically focused on mothers with young children. In some countries, nutritional services are provided directly to adolescent girls because of early marriage and childbearing conditions [37]. In 2016, the Population Council conducted a situation analysis on the ASRH programme which was implemented from 2005 to 2015 and showed that only sixteen programmes out of thirty-two were exclusively focused on adolescents aged 10 to 19 years, among the thirty-two programmes eleven programmes were exclusively focused on girls, and twenty-one programmes focused on both males and females [38]. The Bangladeshi Association for Life Skills, Income, and Knowledge for Adolescents (BALIKA) programme focuses to empowering adolescents, to access on education, decision-making, and economic empowerment to eliminate early marriage [39]. Although it is significant to empower females to make their own decisions on marriage and childbirth, no programme especially focuses on the child undernutrition of adolescent mothers. The Advance Adolescent Health (A2H) programme focuses on the delayed age of marriage, the delay of the first childbirth, and the gap between the first and second births using family planning and sensitising partners [40]. Though ‘Sustainable Development Goal’ SDG-3 focuses on improving health and well-being for people of all ages but study emphasises the effective convergence of interventions to reduce early marriage and birth to reduce all forms of malnutrition [41, 42]. The National Strategy for Adolescent Health 2017–2030 focuses on undernutrition among adolescent girls and boys, reducing pregnancy-related complications and nutritional risk, reducing micronutrient deficiencies, and reducing the risk of overweight and obesity among adolescents [43]. Still, the focus on the under-five children of adolescent mothers remains absent. Though there are several programmes, interventions, and policy initiatives taken to end early marriage by 2030, significant efforts are needed (Fig. 4).

## Discussion

The analysis of the BDHS-2017-18 data revealed several key findings. First, a significant number of mothers in Bangladesh give birth before the age of nineteen. Second, children born to adolescent mothers are more likely to experience undernutrition, including wasting, being underweight, and either form of undernutrition compared to children born to older mothers. Third, we also found that stunting of children by adolescent mothers, was marginally statistically significant. Lastly, factors associated with adolescent motherhood in Bangladesh include a larger age gap (five to ten years or more than ten years) between the husband and wife, as well as the husband’s education level. Moreover, this study is the first is examine to the programme gaps related to adolescent mothers and child nutrition systematically. The results show that approximately 89% of mothers married, and 71% of mothers had their first childbirth during their adolescence (≤ 19 years). The frequency of the mother’s age during first childbirth in Bangladesh is consistent with the findings of the earlier study conducted in Bangladesh [44]. Another study shows relevant findings, 83% of women married and 62% gave birth before 18 years [45]. Additionally, 33% of adolescent mothers marry before the age of 15 years in Bangladesh [46] and 68% of females had a child between the age of 15–19 years [47]. Many girls had children earlier in their 19 years old, which has adverse effects on parenting and raising children. In the USA, adolescent mothers’ experiences with childbearing have an impact on their thoughts, relationships, and emotions [48]. The high proportion of adolescence mothers, defined as those aged 19 or younger, raises concerns about various aspects related to maternal and child health. It is well-known that early motherhood can pose unique challenges and risks for both the mother and the child. These challenges may include inadequate prenatal care, limited access to education, economic constraints, and social stigmatisation. Therefore, the high prevalence of adolescent mothers in Bangladesh calls for targeted interventions and support systems to address the specific needs of this vulnerable population.

The under-five children of adolescent mothers had a prevalence of stunting at 34%, wasting 9.0%, and underweight 24% respectively. In Bangladesh, another study on the MNCH initiatives run by the non-governmental organisation BRAC revealed that among children whose mothers were younger than nineteen years old, the frequency of stunting was 15.9%, wasting was 14.7%, and underweight was 22.4% [49]. This study found lower levels of undernutrition, such as stunting, wasting, and being underweight, as this study was conducted in the implementation area, which may have an impact. Contrast findings were also found in Ghana, where under-5 children of adolescent mothers aged 15 to 19 years have higher risks of being stunted 39.0% and wasted 8.0%.

We found that under-five children of adolescent mothers were at significantly higher risk of wasting, being underweight, and any form of undernutrition, either stunting, wasting, or being underweight. According to this study, there is a higher risk of child undernutrition (wasting, underweight, and any forms of undernutrition) if the first child is born before the age of 19 years or younger. In Bangladesh, one in five children under the age of five suffers from both single and multiple concurrent forms of undernutrition [50]. Adolescent mothers are more likely to have undernourished children, for example, they are shorter and underweight compared to older mothers this finding is consistent with our study findings except, for the stunting of children [12]. A previous study also shows that poor maternal nutrition, low levels of education, limited access to health care, and inadequate supplementary feeding practises, and all linked to undernutrition in children born to adolescent women [12]. Contrast findings also found in a prospective study conducted in LMICs (Brazil, Guatemala, India, the Philippines, and South Africa) showed that adolescent mothers aged ≤ 19 years are linked to stunting in children aged of 2-years [16]. The stunted P value of 0.08 and aOR 1.25 reveal a potential association between the variables however, this may be due to variations in sample size, demographics, cultural practises, socioeconomic status, poor nutrition during pregnancy, and access to healthcare. Additionally, the study design and statistical analysis could also play a role. Further research is warranted to explore these discrepancies and gain a deeper understanding of the complex relationship between adolescent motherhood and stunting [33]. Moreover, this study revealed that children of adolescent mothers have higher risk of undernutrition than those of adult mothers. Moreover, children of adolescent mothers are at least 1.3 times more likely to be underweight, wasted, or any form of underweight than adult mothers. Adolescent pregnancies are associated with a higher risk of undernutrition in India. In addition to being shorter for their age, children of adolescent mothers had a 5% point higher chance of being stunted than children of elderly mothers [51]. According to this study, mothers who gave birth to their first child before the age of 19 had an increased risk for the undernourishment of their children. This may happen to mothers under the age of nineteen, who may lack the maturity to care for their children appropriately. Lack of knowledge about maternity and newborn care, particularly breastfeeding and prenatal care, among many young mothers [52]. Mothers who are under 19 years old and become pregnant may leave school early and do not have any stable income sources. Pregnant females are regularly pushed or pressured to leave school, which negatively affects their possibilities of further education and employment [1]. Because of the early pregnancy, they may provide their children with less care, nursing, and nurturing than adult mothers; this could result in a lack of care, nursing, and nurturing overall [52]. Early parenthood has had a detrimental effect on adolescent girls’ relationships with their spouses, families, schools, and society overall [52]. Because of malnourishment and other growth limitations, this may impact their children’s physical and mental development [53]. The results of our study show that about 88% of females get married before turning 19 years old. Consequently, it is crucial to research the causes of this young marriage age and its effects on several factors, including child stunting and wasting. Further research is needed to understand the causal relationship between adolescent motherhood and child undernutrition.

This study found that a higher spousal age gap and lower education level of the husband increased the risk of mothers’ first childbirth during adolescence. Similar findings were shown in a study conducted in 48 LMICs, in which spousal age difference with the partner is associated with adolescent motherhood among married adolescent girls [54]. This may occur due to unequal power relations and a lack of communication regarding decision-making in the family. According to 2004–2014 data, adolescent women with low education levels, and lower economic status were associated with adolescent birth [47]. Literature suggested that factors related to marriage during adolescence include a lack of gender equality in decision-making, pressure to become pregnant early, limited access to healthcare services, unable to obtain healthcare support, poverty, less education, and inadequate knowledge of maternal and child nutrition as reasons for the adverse outcomes of adolescent mothers’ child health and nutrition [15, 41, 55–57].

From the programmatic perspective, to tackle child undernutrition, messages must be delivered directly to adolescent mothers in both rural and urban regions [37]. Delaying conception can be achieved through family planning programmes for currently married couples or marriage registration contacts [58]. In contrast, well-planned pregnancies among mothers through family planning can lower the incidence of undernutrition in children [59]. Targeting both wives and husbands in maternal nutrition programmes aimed at promoting the adoption of proper dietary behaviours can help achieve significant outcomes [60]. Enhancing maternal dietary diversity, micronutrient supplement consumption, and exclusive breastfeeding practices can be achieved through an established MNCH programme that offers nutrition counselling, community engagement, free supplements, and weight-gain monitoring [61]. Moreover, the MNCH programme can help develop and test interventions to delay pregnancy after adolescence [15]. Girls who are already married should focus on delaying pregnancy. Massive social efforts could effectively encourage family planning and delay conception among adolescent girls [41]. A decomposition analysis shows that 9% of early marriages can be reduced in Bangladesh through the enhancement of women’s education [62]. In Bangladesh, addressing undernutrition in adolescent mothers and children requires a comprehensive and multidimensional approach encompassing education, healthcare access, economic empowerment, and social support. Moreover, it is necessary to strengthen services from adolescent-friendly health corners at the union level to district-level health facilities across the country to empower adolescents regarding sexual and reproductive health.

### Recommendations

From our study results policymakers and programme implementers can identify the programmatic gaps and they will design an effective program focusing on adolescent mothers and their child undernutrition. Moreover, this study will help to understand the importance of promoting comprehensive sex education; empowering adolescent married girls through education and skill training; and engaging men and boys in discussing reproductive health and gender equality; effective interventions and policies are necessary to delay adolescent pregnancy and child nutrition. Similarly, it also needs to emphasis the married adolescents who have a higher age gap with husbands more than five years and above to reduce high rates of early pregnancy and risk of being undernourished. In contrast, from this study results, we can break the cycle of child undernutrition and its negative consequences by enhancing access to healthcare, nutritional counselling, and comprehensive mother and child welfare programmes by engaging healthcare professionals, policymakers, researchers, programme implementers, and community leaders. Additionally, to understand the factors behind the high proportion of adolescent mother qualitative research is required to understand the factors behind the high proportion of adolescent mothers and a prospective cohort study is required to understand the causal relationship between adolescent motherhood and child undernutrition.

### Strengths and limitations

This study has some strengths and limitations. First, this study provides insights into a comprehensive understanding of the mother age during the first childbirth and the undernutrition of children aged under-5 years- based on nationally representative demographic and health survey data. Second, national-level estimates will help governments, policymakers, donor organisations, and other non-governmental organisations to implement targeted intervention programmes. Third, this analysis represents the result from a representative sample size collected from different regions of Bangladesh. This study also has some limitations. First, the BDHS-17-18 data are five years old, but this are the most updated national representative data until the BDHS-2022 data is published. Second, the association between the mother first childbirth during adolescence and the undernutrition of children aged below five years has been demonstrated, even though this cross-sectional methodology is inappropriate for examining causal relationships. Third, the recall process was used to collect data on mothers who gave birth before the age of 19 years, increasing the possibility of recall errors. Finally, in this analysis, we examined the association between mothers’ first childbirth during ≤ 19 years with the undernutrition of children, however, we did not examine the association of mother’s first childbirth during < 15 years with their child’s undernutrition, which is a limitation to obtain a comprehensive understanding.

## Conclusion

The findings emphasise the undernutrition of under-five children, particularly those born to adolescent mothers’ aged ≤ 19 years. Public health measures, educational programmes, and support networks need to be adapted to the unique requirements of first-time adolescent mothers. Moreover, continued research and liaison with donors and partner organisations are important for establishing effective ways to support the healthy growth and development of children born to adolescent mothers and mothers of all ages. Finally, investing in the health and well-being of adolescent mothers and their children is crucial for the healthy future and prospects of the nation.

### Electronic supplementary material

Below is the link to the electronic supplementary material.

Supplementary Material 1

Supplementary Material 2

## Data Availability

Available at: https://dhsprogram.com/data/dataset_admin/index.cfmThe DHS data sets are accessible to the public and anyone can obtain data set upon valid request and using the user login and password. The DHS program does not have a direct link to access the data.

## Abbreviations

LMICs: Low-and Middle-Income Countries
BALIKA: Bangladeshi Association for Life Skills, Income, and Knowledge for Adolescents
A2H: Advance Adolescent Health
ASRH: Adolescent Sexual and Reproductive Health
IYCN: Infant and Young Child Nutrition
SDG: Sustainable Development Goal
MNCH: Maternity, Neonatal, and Child Health
BMI: Body Mass Index
