# Supplementary material for: Understanding the socio-demographic and programmatic factors associated with adolescent motherhood and its association with child undernutrition in Bangladesh
Source: BMC Public Health. 2024 Aug 13;24:2200. doi: 10.1186/s12889-024-19355-3 (PMC11321164; doi:10.1186/s12889-024-19355-3)
Supplement: Supplementary file 1 — Supplementary Material 1 [file 12889_2024_19355_MOESM1_ESM.docx]

**Supplementary Information:**

**Supplementary Table 1: Association of mother's first childbirth during adolescence with their child’s undernutrition**

| **Variable** | **Wasted** | | **Stunted** | | **Underweighted** | |
| --- | --- | --- | --- | --- | --- | --- |
|  | **Number (%)** | **P value** | **Number (%)** | **P value** | **Number (%)** | **P value** |
| **Mothers’ age at first childbirth** | | | | | | |
| ≤19 years | 483 (9.0) | 0.008* | 1805 (33.7) | <0.001* | 1271 (23.7) | <0.001* |
| >19 years | 164 (7.2) |  | 587 (25.7) |  | 425 (18.6) |  |
| **Mother’s BMI** | | | | | | |
| Underweight | 158 (14.0) | <0.001* | 474 (42.0) | <0.001* | 373 (33.1) | <0.001* |
| Normal weight | 366 (8.1) |  | 1471 (32.4) |  | 1018 (22.4) |  |
| Overweight/  Obese | 123 (10.7) |  | 447 (42.5) |  | 305 (28.0) |  |
| **Mother’s education** | | | | | | |
| No formal education | 66 (12.3) | <0.001* | 244 (45.4) | <0.001* | 198 (36.9) | <0.001* |
| Primary | 198 (8.9) |  | 872 (39.5) |  | 612 (27.7) |  |
| Secondary | 308 (8.5) |  | 1085 (30.0) |  | 752 (20.8) |  |
| Higher | 75 (5.9) |  | 191 (14.9) |  | 134 (10.5) |  |
| **Delivery via C- section** | | | | | | |
| Yes | 103 (6.6) | 0.006* | 356 (22.7) | <0.001* | 218 (13.9) | <0.001* |
| No | 280 (8.9) |  | 1114 (35.3) |  | 717 (22.7) |  |
| **ANC visits** | | | | | | |
| ≤ 4 | 249 (8.6) | 0.226 | 950 (32.9) | <0.001* | 625 (21.7) | <0.001* |
| > 4 | 125 (7.6) |  | 436 (26.5) |  | 263 (16.0) |  |
| **Types of household head** | | | | | | |
| Self | 40 (7.6) | 0.017* | 150 (28.4) | <0.001* | 99 (18.9) | <0.001* |
| Husband | 402 (9.1) |  | 1460 (33.2) |  | 1072 (24.4) |  |
| Father | 73 (8.3) |  | 252 (28.5) |  | 175 (19.8) |  |
| Fathers-in-law | 92 (6.4) |  | 409 (28.6) |  | 261 (18.2) |  |
| Others | 40 (9.9) |  | 121 (30.2) |  | 89 (22.2) |  |
| **Currently working status** | | | | | | |
| Working | 265 (8.6) | 0.769 | 1057 (34.2) | <0.001* | 745 (24.1) | <0.001* |
| Not working | 382 (8.4) |  | 1335 (29.3) |  | 951 (20.9) |  |
| **Father’s education** | | | | | | |
| No formal education | 114 (10.00) | 0.013* | 495 (43.4) | <0.001* | 355 (31.1) | <0.001* |
| Primary | 227 (8.6) |  | 988 (37.6) |  | 676 (25.7) |  |
| Secondary | 215 (8.7) |  | 687 (27.8) |  | 497 (20.1) |  |
| Higher | 91 (6.5) |  | 222 (15.9) |  | 168 (12.0) |  |
| **Wealth index** | | | | | | |
| Poorest | 171 (10.0) | 0.058 | 702 (41.1) | <0.001* | 498 (29.1) | <0.001* |
| Poorer | 131 (8.5) |  | 596 (38.6) |  | 408 (26.4) |  |
| Middle | 113 (8.2) |  | 426 (30.9) |  | 300 (21.8) |  |
| Richer | 127 (8.3) |  | 419 (27.4) |  | 310 (20.3) |  |
| Richest | 105 (7.1) |  | 249 (16.8) |  | 180 (12.1) |  |
| **Childbirth weight** | | | | | | |
| Low birth weight (<2500 gm) | 32 (9.8) | 0.076 | 137 (41.8) | <0.001* | 107 (32.6) | <0.001* |
| Normal weight (≥ 2500 gm) | 126 (6.9) |  | 382 (21.1) |  | 225 (12.4) |  |
| **Age of children** | | | | | | |
| <24 **months** | 265 (8.2) | 0.389 | 900 (27.6) | <0.001* | 570 (17.5) | <0.001* |
| ≥ 24 **months** | 382 (8.7) |  | 1492 (33.9) |  | 1126 (25.7) |  |
| **Sex of children** | | | | | | |
| Male | 363 (9.1) | 0.035* | 1253 (31.4) | 0.773 | 876 (21.9) | 0.648 |
| Female | 284 (7.8) |  | 1139 (31.1) |  | 820 (22.4) |  |

* Significant P values

**Supplementary Table 2: Associated factors of mothers' first childbirth during adolescence**

| **Characteristics** | **>19 (n= 2,287)** | **<=19 (n=5,356)** | **P- value** |
| --- | --- | --- | --- |
| **Spousal age gap (n= 7,638)** | | | |
| <5 years | 715 (31.26) | 1,186 (22.14) | <0.001* |
| 5- 10 years | 1,078 (47.14) | 2802 (52.32) |  |
| >10 years | 494 (21.60) | 1,368 (25.54) |  |
| **Mothers’ employment status (7,640)** | | | |
| Not working | 1392 (60.87) | 2,986 (55.75) | <0.001* |
| Working | 895 (39.13) | 2370 (44.25) |  |
| **Partners education level** | | | |
| No formal education | 252 (11.02) | 888 (16.58) | <0.001* |
| Primary | 539 (23.57) | 2,091 (39.04) |  |
| Secondary | 699 (30.56) | 1,774 (33.12) |  |
| Higher | 797 (34.85) | 603 (11.26) |  |
| **Media exposure** | | | |
| Not at all | 627 (27.42) | 2,190 (40.89) | <0.001* |
| Less than once a week | 200 (8.75) | 569 (10.62) |  |
| At least once a week | 1,460 (63.84) | 2,597 (48.49) |  |
| **Place of residence** | | | |
| Urban | 974 (42.59) | 1,622 (30.28) | <0.001* |
| Rural | 1,313 (57.41) | 3,734 (69.72) |  |
| **Mothers education level** | | | |
| No formal education | 124 (5.42) |  | <0.001* |
| Primary | 429 (18.76) | 1,780 (33.23) |  |
| Secondary | 911 (39.83) | 2,708 (50.56) |  |
| Higher | 823 (35.99) | 455 (8.50) |  |
| **Wealth index** | | | |
| Poorest | 312 (13.64) | 1,397 (26.08) | <0.001* |
| Poorer | 333 (14.56) | 1,211 (22.61) |  |
| Middle | 385 (16.83) | 992 (18.52) |  |
| Richer | 501 (21.91) | 1,027 (19.17) |  |
| Richest | 756 (33.06) | 729 (13.61) |  |
| **Administrative Division** | | | |
| Barisal | 222 (9.71) | 579 (10.81) | <0.001* |
| Chattogram | 334 (14.60) | 895 (16.71) |  |
| Dhaka | 384 (16.79) | 692 (12.92) |  |
| Khulna | 250 (10.93) | 559 (10.44) |  |
| Mymensingh | 258 (11.28) | 653 (12.19) |  |
| Rajshahi | 202 (8.83) | 595 (11.11) |  |
| Rangpur | 221 (9.66) | 662 (12.36) |  |
| Sylhet | 416 (18.19) | 721 (13.46) |  |

* Significant P values
