# Supplementary material for: Understanding the socio-demographic and programmatic factors associated with adolescent motherhood and its association with child undernutrition in Bangladesh
Source: BMC Public Health. 2024 Aug 13;24:2200. doi: 10.1186/s12889-024-19355-3 (PMC11321164; doi:10.1186/s12889-024-19355-3)
Supplement: Supplementary file 2 — Supplementary Material 2 [file 12889_2024_19355_MOESM2_ESM.docx]

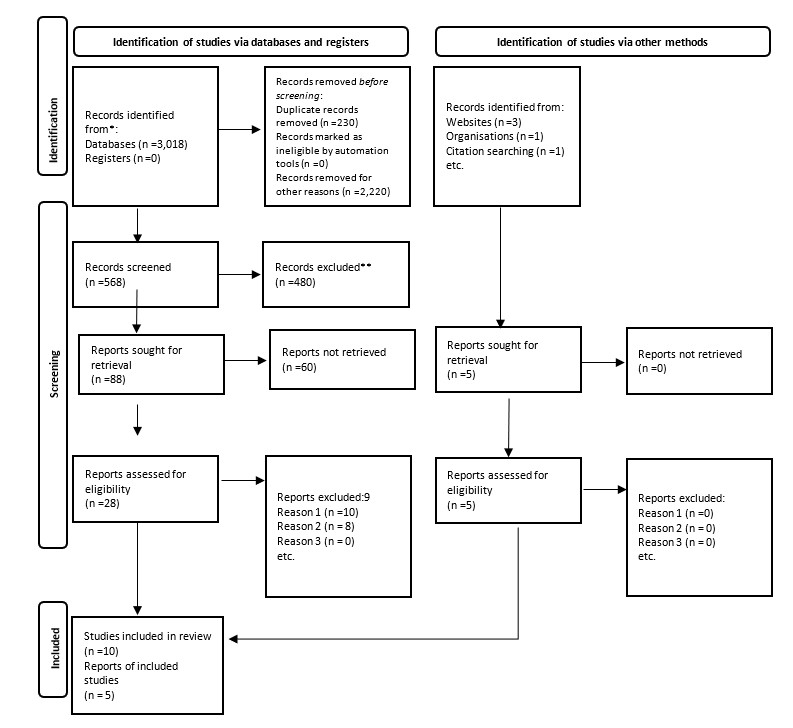


**Supplementary Figure 1: Flow diagram of the literature review process flowing PRISMA guideline**
